# The Relationship Between Glycated Albumin and Time in Tight Range in Type 2 Diabetes

**DOI:** 10.1111/1753-0407.70073

**Published:** 2025-03-26

**Authors:** Jiaying Ni, Wenshuo Han, Yaxin Wang, Jiamin Yu, Wei Lu, Yufei Wang, Xiaojing Ma, Jingyi Lu, Jian Zhou

**Affiliations:** ^1^ Department of Endocrinology and Metabolism, Shanghai Sixth People's Hospital Affiliated to Shanghai Jiao Tong University School of Medicine; Shanghai Clinical Center for Diabetes Shanghai Diabetes Institute; Shanghai Key Laboratory of Diabetes Mellitus Shanghai China

**Keywords:** glycated albumin, time in tight range, type 2 diabetes

## Abstract

**Aims:**

Among the new glucose metrics derived from continuous glucose monitoring, the concept of time in tight range (TITR) has gained increasing attention. We aimed to assess the association between TITR and traditional glycemic indicators, such as glycated albumin (GA).

**Methods:**

A total of 310 patients with type 2 diabetes on a stable glucose‐lowering regimen over the previous 3 months were enrolled. TITR and time in range (TIR) were calculated using continuous glucose monitoring data collected over a minimum of 5 days. Spearman correlation analysis was performed to assess the relationships between traditional glycemic indicators, including GA and HbA1c, with TITR and TIR. Receiver operating characteristic curves were used to evaluate the predictive value of GA for TITR > 50% and TIR > 70%.

**Results:**

The median levels of GA and HbA1c were 15.6% (14.0%, 17.3%) and 6.5% (6.1%, 7.1%), respectively. Median TITR and TIR were 70.0% (56.0%, 81.0%) and 91.0% (84.0%, 96.8%), respectively. Spearman correlation analysis showed a moderate negative relationship between GA and both TITR and TIR. The optimal GA cutoff for identifying either TITR > 50% or TIR > 70% was 17.4%. Moreover, combining GA with fasting plasma glucose or 2‐h postprandial glucose significantly enhanced the ability to identify TITR > 50%, achieving performance comparable to the combination of HbA1c and plasma glucose.

**Conclusions:**

In patients with type 2 diabetes, a GA cutoff of 17.4% effectively identifies TITR > 50%.

**Summary**

- Glycated albumin (GA) at a threshold of 17.4% was found to effectively identify type 2 diabetes patients with time in tight range (TITR) > 50%.
- Combining GA with fasting or postprandial plasma glucose significantly improved the identification efficacy for TITR > 50%.

## 1 Introduction

Time in range (TIR), defined as the percentage of time spent within the target glucose range of 3.9–10.0 mmol/L, is a key metric derived from continuous glucose monitoring (CGM) and has been widely used for the assessment of glucose control [1, 2, 3]. In recent years, the rise of new technologies and therapies, including advanced hybrid closed‐loop automated insulin delivery systems and new hypoglycemic drugs, has made it possible to achieve better glycemic control [4, 5]. Building on this concept, time in tight range (TITR), which refers to the percentage of time glucose remains within the narrower range of 3.9–7.8 mmol/L, has been proposed as a more refined clinical trial endpoint for CGM [6], as it more closely mirrors the physiological glucose levels seen in healthy individuals. Studies have indicated that for well‐controlled persons with diabetes, especially those with stricter targets for average glucose and glycated hemoglobin A1c (HbA1c), TITR provides a clearer view of fluctuations outside the target range compared to TIR, offering more precise insights into their glycemic control [7]. Additionally, our previous study found that TITR continued to exhibit a significant correlation with diabetic retinopathy even in the TIR > 70% subgroup [8].

However, despite the continuous and comprehensive blood glucose monitoring provided by CGM, its clinical application remains limited due to factors such as high costs, inadequate insurance coverage, and insufficient education and awareness, which may lead to hesitancy among some patients to utilize the technology [9, 10]. Traditional blood glucose monitoring indicators, such as HbA1c and GA, are commonly used in clinical practice to reflect average blood glucose levels over the past 2–3 months and 2–3 weeks, respectively. GA is not influenced by factors like hemoglobin variants or pregnancy, making it a reliable complement to HbA1c in glycemic assessment [3].

Research on the relationship between GA and CGM metrics has been a subject of ongoing interest [11, 12, 13]. One study found that GA had a stronger correlation with TIR than HbA1c and was the most accurate predictor of TIR over an 8‐week period [11]. However, to date, no studies have directly explored the relationship between GA and TITR, nor have they established corresponding cutoffs for achieving TITR targets, particularly in patients with type 2 diabetes.

Therefore, this study aims to explore the relationship between GA and TITR in well‐controlled patients, comparing the findings with HbA1c to provide further insights into the role of traditional blood glucose monitoring indicators in glycemic assessment.

## 2 Materials and Methods

### 2.1 Study Population

This study recruited patients with type 2 diabetes who underwent complications screening and wore CGM during hospitalization in the Department of Endocrinology and Metabolism at Shanghai Sixth People's Hospital Affiliated to Shanghai Jiao Tong University School of Medicine from May 2020 to July 2023. They were included if they voluntarily participated in the study and met the following criteria: (a) aged 18 years or older with the presence of type 2 diabetes [14]; (b) an acceptable glycemic control level of HbA1c ≤ 8.0% [1]; (c) with a stable treatment regimen over the past 3 months; (d) available clinical data, including HbA1c, GA, and CGM metrics. Major exclusion criteria included: (a) recent or current diabetic ketoacidosis; (b) ongoing glucocorticoid therapy; (c) pregnancy, malignancy, or psychiatric disorders; (d) CGM usage for fewer than 5 days, as studies demonstrated that TIR derived from at least 5 days of CGM data strongly correlated with 3 months of CGM data (*R* ^2^ > 0.80) [15].

The study adhered to the principles of the Declaration of Helsinki and received ethical approval from the Research Ethics Committees of Shanghai Sixth People's Hospital affiliated with Shanghai Jiao Tong University School of Medicine. Written informed consent was obtained from all participants.

### 2.2 Anthropometric and Biochemical Assessment

All participants completed standardized questionnaires to collect information on age, sex, diabetes duration, and prescribed medications. Comprehensive physical examinations were performed, including height, weight, and blood pressure, with body mass index calculated. Blood pressure was recorded three times using a standard mercury sphygmomanometer after 5 min of seated rest, and the average of the readings was used for analysis. After a 10‐h overnight fast, venous blood samples were collected. Fasting plasma glucose (FPG), HbA1c, GA, serum creatinine, hemoglobin, and serum albumin were measured using previously described standard methods [16]. Postprandial glucose at 2 h (2hPG) was assessed using a mixed‐meal test. GA was measured via an enzyme‐based assay kit (Lucica GA‐L, Asahi Kasei Pharma, Tokyo, Japan) on a 7600 autoanalyzer (Hitachi, Tokyo, Japan) with intra‐ and interassay coefficients of variation of 1.47%–3.30% and 1.95%–4.73%, respectively. HbA1c was detected by high‐performance liquid chromatography (Bio‐Rad D100, Hercules, CA, USA). The estimated glomerular filtration rate (eGFR) was calculated according to the Chronic Kidney Disease Epidemiology equation [17]. The diagnostic criteria for diabetic nephropathy, diabetic retinopathy, carotid atherosclerosis, and lower‐extremity atherosclerotic disease were as described previously [18, 19, 20, 21].

### 2.3 Metrics Derived From CGM

A retrospective CGM system (iPro 2, Medtronic Inc) was used for subcutaneous interstitial glucose monitoring. The sensors of the CGM systems were inserted on the first day of hospital admission and then automatically generated a daily record of 288 glucose values. For the calibration of the CGM systems, capillary blood glucose values were measured at least once every 12 h using a SureStep blood glucose meter (LifeScan). As previously described [21], mean sensor glucose (MSG), standard deviation (SD), and coefficient of variation (CV) were calculated, with SD and CV serving as indicators of glycemic variability. TIR was defined as the average percentage of time for glucose levels between 3.9 and 10.0 mmol/L during a 24‐h period, while TITR was defined as the percentage of time within 3.9–7.8 mmol/L. Additionally, the percent time spent in the glucose ranges of > 13.9 mmol/L (TAR_> 13.9_), > 10.0 mmol/L (TAR_> 10.0_), > 7.8 mmol/L (TAR_> 7.8_), < 3.9 mmol/L (TBR_< 3.9_), and < 3.0 mmol/L (TBR_< 3.0_) were calculated. Participants adhered to a standardized diet, with daily caloric intake restricted to 25 kcal/kg (1 kcal = 4.18 kJ), comprising 55% carbohydrates, 17% protein, and 28% fat.

### 2.4 Statistical Analysis

Statistical analyses were performed by SPSS version 26.0 (SPSS Inc., Chicago, IL, USA) and R version 4.2.2 (RStudio Inc., Boston, MA, USA). Continuous variables with a normal or non‐normal distribution were presented as mean ± SD or median (25th percentile, 75th percentile), respectively, and categorical variables as *n* (%). Based on previous studies [2, 22], TITR and TIR were categorized as binary variables using cutoff points of 50% and 70%, respectively. Similarly, CV was categorized as a binary variable using a 33% cutoff [23]. Spearman correlation analysis was conducted to evaluate relationships between traditional glycemic indicators and CGM metrics. A prior study by our group demonstrated that glycemic variability assessed by CV significantly impacts the correlation between TIR and glucose management indicator [24]. Therefore, partial correlation analysis was performed to further adjust for CV and assess the stability of the relationships between GA, HbA1c, and TITR or TIR. Receiver operating characteristic (ROC) curves were plotted to explore the ability of various glycemic indicators to distinguish TITR > 50% or TIR > 70%. The optimal cutoffs were determined using the Youden index. Differences between ROC curves were compared using the DeLong test. The sensitivity and specificity of different glycemic indicators for distinguishing TITR > 50% or TIR > 70% were compared using McNemar's test. The integrated discrimination improvement (IDI) and net reclassification improvement (NRI) indices were calculated to compare the discrimination and classification ability between GA and other glycemic metrics. Sensitivity analysis was performed after excluding factors that could influence HbA1c and GA measurements, such as anemia, hypoproteinemia, and liver cirrhosis. All *p* values were two‐sided, with *p* < 0.05 considered statistically significant.

## 3 Results

### 3.1 Clinical Characteristics of Study Participants

A total of 310 patients with type 2 diabetes were included in this study (Figure S1), with clinical characteristics detailed in Table 1. The median age of the study population was 60.0 (52.0, 66.0) years, with a median diabetes duration of 10.0 (5.0, 15.0) years. The median HbA1c level was 6.5% (6.1%, 7.1%), and the median GA level was 15.6% (14.0%, 17.3%). The average CGM wearing duration was 6.0 (5.0, 7.0) days, with median TIR and TITR levels of 91.0% (84.0%, 96.8%) and 70.0% (56.0%, 81.0%), respectively. Treatment regimens included lifestyle interventions alone for 28.7% of patients, oral antidiabetic drugs for 54.2%, injectable antidiabetes medication alone for 2.9%, and combined therapy for 14.2%.

**TABLE 1:** Characteristics of the participants.

	Total
Clinical characteristics	
*N*.	310
Male, *n* (%)	162 (52.3%)
Age, years	60.0 (52.0, 66.0)
Diabetes duration, years	10.0 (5.0, 15.0)
Systolic blood pressure, mmHg	130.5 (120.0, 141.8)
Diastolic blood pressure, mmHg	80.0 (72.2, 85.0)
Body mass index, kg/m^2^	24.0 (22.3, 26.6)
eGFR, mL/min/1.73 m^2^	94.9 (85.4, 101.3)
Fasting plasma glucose, mmol/L	5.8 (5.1, 6.6)
2‐h plasma glucose, mmol/L	8.4 (6.6, 10.6)
GA, %	15.6 (14.0, 17.3)
HbA1c, %	6.5 (6.1, 7.1)
Diabetic nephropathy^a^, *n* (%)	58 (22.7%)
Diabetic retinopathy^b^, *n* (%)	65 (25.9%)
Carotid atherosclerosis^c^, *n* (%)	151 (52.1%)
Lower‐extremity atherosclerotic disease^c^, *n* (%)	121 (41.7%)
Glucose‐lowering regimens	
Lifestyle intervention only, *n* (%)	89 (28.7%)
Oral antidiabetes medication only, *n* (%)	168 (54.2%)
Metformin, *n* (%)	116 (69.0%)
Sulfonylureas, *n* (%)	29 (17.3%)
Thiazolidinediones, *n* (%)	3 (1.8%)
Glinides, *n* (%)	10 (6.0%)
Dipeptidyl peptidase 4 inhibitors, *n* (%)	63 (37.5%)
α‐glucosidase inhibitors, *n* (%)	60 (35.7%)
Sodium‐glucose cotransporter 2 inhibitors, *n* (%)	68 (40.5%)
Injectable antidiabetes medication only, *n* (%)	9 (2.9%)
Combined antidiabetes medication, *n* (%)	44 (14.2%)
Other medications	
Antihypertension agents, *n* (%)	134 (43.2%)
Lipid‐lowering agents, *n* (%)	190 (61.3%)
CGM metrics	
Number of days CGM worn, days	6.0 (5.0, 7.0)
Mean sensor glucose, mmol/L	7.2 (6.7, 8.0)
SD of mean glucose, mmol/L	1.7 (1.3, 2.1)
CV, %	22.9 (18.6, 27.7)
TAR_> 13.9_, %	0.0 (0.0, 1.0)
TAR_> 10.0_, %	8.0 (2.0, 15.0)
TAR_> 7.8_, %	29.0 (18.0, 43.8)
TITR, %	70.0 (56.0, 81.0)
TIR, %	91.0 (84.0, 96.8)
TBR_< 3.9_, %	0.0 (0.0, 1.0)
TBR_< 3.0_, %	0.0 (0.0, 0.0)

*Note:* Data were expressed as median (interquartile range) or number (percentage).

Abbreviations: CGM, continuous glucose monitoring; CV, coefficient of variation; eGFR, estimated glomerular filtration rate; GA, glycated albumin; HbA1c, glycated hemoglobin A1c; SD, standard deviation; TAR, time above range; TBR, time below range; TIR, time in range (3.9–10.0 mmol/L); TITR, time in tight range (3.9–7.8 mmol/L).

^a^ The number of participants included is 255.

^b^ The number of participants included is 251.

^c^ The number of participants included is 290.

### 3.2 The Relationship Between GA and TITR

Spearman correlation analysis revealed a moderate negative correlation between GA and TITR (*r* = −0.437, *p* < 0.001) (Table 2). After adjusting for CV, partial correlation analysis confirmed a persistent negative correlation between GA and TITR (*r* = −0.374, *p* < 0.001). When stratified by CV target, no significant difference was found in the slopes of the regression lines for GA and TITR (*p* for interaction > 0.05). Similarly, HbA1c was also moderately negatively correlated with TITR (*r* = −0.500, *p* < 0.001), and this relationship was not affected by CV (Table 2).

**TABLE 2:** Correlation of GA and HbA1c with clinical characteristics and CGM metrics.

	GA				HbA1c			
	*r*	*p*	*r'*	*p*	*r*	*p*	*r'*	*p*
Clinical characteristics								
Age	0.187	0.001			0.034	0.549		
Diabetes duration	0.249	< 0.001			0.086	0.130		
Systolic blood pressure	0.139	0.014			0.193	0.001		
Diastolic blood pressure	−0.029	0.608			0.103	0.071		
Body mass index	−0.196	0.001			0.076	0.183		
eGFR	−0.088	0.122			0.021	0.715		
Fasting plasma glucose	0.375	< 0.001			0.530	< 0.001		
2‐h plasma glucose	0.379	< 0.001			0.475	< 0.001		
GA	—	—			0.555	< 0.001		
HbA1c	0.555	< 0.001			—	—		
CGM metrics								
Mean sensor glucose	0.447	< 0.001	0.410	< 0.001	0.561	< 0.001	0.544	< 0.001
SD of mean glucose	0.381	< 0.001	0.403	< 0.001	0.268	< 0.001	0.517	< 0.001
CV	0.246	< 0.001	—	—	0.062	0.275	—	—
TAR_> 13.9_	0.305	< 0.001	0.179	0.002	0.251	< 0.001	0.223	< 0.001
TAR_> 10.0_	0.457	< 0.001	0.350	< 0.001	0.433	< 0.001	0.422	< 0.001
TAR_> 7.8_	0.443	< 0.001	0.386	< 0.001	0.524	< 0.001	0.501	< 0.001
TITR	−0.437	< 0.001	−0.374	< 0.001	−0.500	< 0.001	−0.487	< 0.001
TIR	−0.419	< 0.001	−0.322	< 0.001	−0.353	< 0.001	−0.381	< 0.001
TBR_< 3.9_	−0.043	0.446	−0.194	0.001	−0.167	0.003	−0.252	< 0.001
TBR_< 3.0_	0.012	0.835	−0.114	0.045	−0.016	0.784	−0.100	0.081

*Note: r* indicates the spearman correlation of GA and HbA1c with clinical characteristics and CGM metrics. *r'* indicates the partial correlation of GA and HbA1c with CGM metrics after adjusting for CV.

Abbreviations: CGM, continuous glucose monitoring; CV, coefficient of variation; eGFR, estimated glomerular filtration rate; GA, glycated albumin; HbA1c, glycated hemoglobin A1c; SD, standard deviation; TAR, time above range; TBR, time below range; TIR, time in range (3.9–10.0 mmol/L); TITR, time in tight range (3.9–7.8 mmol/L).

### 3.3 Cutoff of GA for Identifying TITR > 50%

**FIGURE 1:**
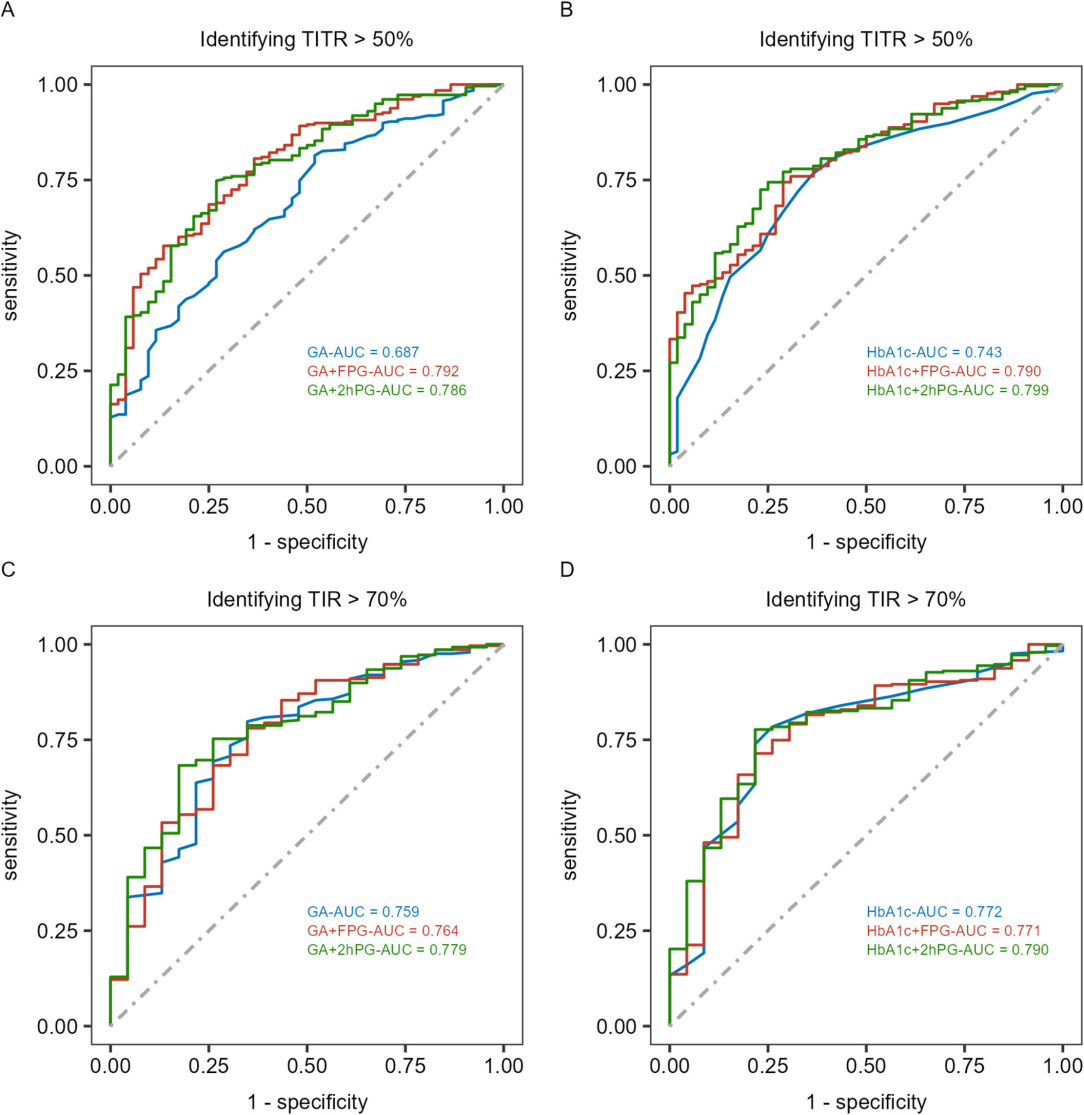
ROC curves of GA, HbA1c and their combinations with FPG or 2hPG in identifying TITR > 50% or TIR > 70%. 2hPG, 2‐h plasma glucose; FPG, fasting plasma glucose; GA, glycated albumin; HbA1c, glycated hemoglobin A1c; ROC, receiver operating characteristic; TIR, time in range (3.9–10.0 mmol/L); TITR, time in tight range (3.9–7.8 mmol/L).

When using GA alone to identify TITR > 50%, the optimal cutoff was 17.4%, with an area under curve (AUC) of 0.687 (Table 3). Combining GA with FPG and 2hPG further improved the identification performance, with AUC values of 0.792 and 0.786, and specificity increasing to 86.54% and 73.08%, respectively (all *p* < 0.05) (Figure 1). Similarly, indicators of discrimination ability, including the IDI and NRI, showed that the combination of GA and FPG/2hPG provided a higher value than GA alone in discrimination for TITR > 50% (Table 3). Compared to GA, the cutoff for HbA1c to identify TITR > 50% was 6.9%. There was no significant difference in performance between GA and HbA1c, and the combination of GA with FPG or 2hPG demonstrated similar effectiveness to the combination of HbA1c with FPG or 2hPG (all *p* > 0.05) (Table 3).

**TABLE 3:** Effectiveness of GA, HbA1c, and their combinations with FPG or 2hPG in identifying TITR > 50%.

TITR > 50%	AUC	Cutoff	Sensitivity (%)	Specificity (%)	PPV (%)	NPV (%)	NRI (95% CI)	IDI (95% CI)
GA	0.687 (0.609, 0.765)	17.4%	81.40	48.08	88.61	34.25	Reference	Reference
HbA1c	0.743 (0.671, 0.815)	6.9%	76.74	63.46	91.24	35.48	0.50 (0.22–0.77)^d^	0.03 (−0.01–0.08)
FPG	0.758 (0.682, 0.835)	6.6 mmol/L	83.72	65.38	92.31	44.74	0.45 (0.17–0.73)^d^	0.10 (0.03–0.18)^e^
2hPG	0.765 (0.698, 0.833)	8.6 mmol/L	59.30^b^	82.69^c^	94.44	29.05	0.28 (−0.01–0.56)	0.06 (−0.00–0.12)
GA + FPG	0.792 (0.728, 0.856)^a^		57.75^b^	86.54^c^	95.51	29.22	0.80 (0.53–1.06)^d^	0.13 (0.07–0.19)^e^
GA + 2hPG	0.786 (0.721, 0.850)^a^		74.81	73.08^c^	93.24	36.89	0.52 (0.24–0.80)^d^	0.09 (0.05–0.13)^e^
HbA1c + FPG	0.790 (0.729, 0.851)^a^		74.42	71.15^c^	92.75	35.92	0.67 (0.40–0.94)^d^	0.12 (0.05–0.19)^e^
HbA1c + 2hPG	0.799 (0.739, 0.859)^a^		74.42	75.00^c^	93.66	37.14	0.55 (0.27–0.83)^d^	0.10 (0.04–0.16)^e^

Abbreviations: 2hPG, 2‐h plasma glucose; AUC, area under the curve; CI, confidence interval; FPG, fasting plasma glucose; GA, glycated albumin; HbA1c, glycated hemoglobin A1c; IDI, integrated discrimination improvement; NPV, negative predict value; NRI, net reclassification improvement; PPV, positive predict value; TITR, time in tight range (3.9–7.8 mmol/L).

^a^ *p* < 0.05 for comparisons of AUC between different models in identifying TITR > 50% compared to GA as the reference indicator.

^b^ *p* < 0.05 for comparisons of sensitivity between different models in identifying TITR > 50% compared to GA as the reference indicator.

^c^ *p* < 0.05 for comparisons of specificity between different models in identifying TITR > 50% compared to GA as the reference indicator.

^d^ *p* < 0.05 for comparisons of NRI between different models in identifying TITR > 50% compared to GA as the reference indicator.

^e^ *p* < 0.05 for comparisons of IDI between different models in identifying TITR > 50% compared to GA as the reference indicator.

### 3.4 The Relationship Between GA and TIR

Similar to its relationship with TITR, both GA and HbA1c showed moderate negative correlations with TIR (*r* = −0.419, *r* = −0.353; both *p* < 0.001), and this relationship was not influenced by CV. When using GA and HbA1c alone to identify TIR > 70%, the optimal cutoffs were 17.4% and 7.0%, with AUC values of 0.759 and 0.772, respectively. There was no statistically significant difference in the identification performance between GA and HbA1c (*p* > 0.05) (Table 4). Additionally, combining GA or HbA1c with FPG or 2hPG did not further improve the identification efficacy (all *p* > 0.05) (Table 4, Figure 1).

**TABLE 4:** Effectiveness of GA, HbA1c, and their combinations with FPG or 2hPG in identifying TIR > 70%.

TIR > 70%	AUC	Cutoff	Sensitivity (%)	Specificity (%)	PPV (%)	NPV (%)	NRI (95% CI)	IDI (95% CI)
GA	0.759 (0.653, 0.864)	17.4%	79.79	65.22	96.62	20.55	Reference	Reference
HbA1c	0.772 (0.673, 0.872)	7.0%	78.40	73.91	97.40	21.52	0.07 (−0.35–0.50)	−0.01 (−0.06–0.04)
FPG	0.582 (0.443, 0.722)^a^	6.6 mmol/L	77.35	47.83	94.87	14.47	−0.52 (−0.94−0.10)^c^	−0.05 (−0.11–0.01)
2hPG	0.697 (0.586, 0.808)	8.9 mmol/L	57.14^b^	78.26	97.04	12.77	−0.22 (−0.65–0.20)	−0.04 (−0.09–0.02)
GA + FPG	0.764 (0.658, 0.870)		78.05	65.22	96.55	19.23	0.24 (−0.18–0.66)	0.01 (−0.02–0.04)
GA + 2hPG	0.779 (0.684, 0.874)		68.29^b^	82.61	98.00	17.27	0.30 (−0.12–0.73)	0.02 (−0.01–0.05)
HbA1c + FPG	0.771 (0.672, 0.871)		71.43	78.26	97.62	18.00	0.19 (−0.24–0.61)	−0.01 (−0.06–0.04)
HbA1c + 2hPG	0.790 (0.701, 0.878)		77.70	78.26	97.81	21.95	0.20 (−0.22–0.62)	−0.00 (−0.05–0.05)

Abbreviations: 2hPG, 2‐h plasma glucose; AUC, area under curve; CI, confidence interval; FPG, fasting plasma glucose; GA, glycated albumin; HbA1c, glycated hemoglobin A1c; IDI, integrated discrimination improvement; NPV, negative predict value; NRI, net reclassification improvement; PPV, positive predict value; TIR, time in range (3.9–10.0 mmol/L).

^a^ *p* < 0.05 for comparisons of AUC between different models in identifying TIR > 70% compared to GA as the reference indicator.

^b^ *p* < 0.05 for comparisons of sensitivity between different models in identifying TIR > 70% compared to GA as the reference indicator.

^c^ *p* < 0.05 for comparisons of NRI between different models in identifying TIR > 70% compared to GA as the reference indicator.

### 3.5 Sensitivity Analysis

After excluding factors that may affect the measurement of GA and HbA1c (Figure S1), the moderate negative correlation between GA and TITR remained significant in 251 patients with type 2 diabetes (*p* < 0.001). The results for identifying TITR > 50% using GA alone or in combination with FPG or 2hPG were consistent with those observed in the overall population (Table S1, Figure S2).

## 4 Discussion

In 310 well‐controlled patients with type 2 diabetes on stable treatment regimens, we observed a significant inverse relationship between GA and TITR, with an optimal cutoff of 17.4% for identifying TITR > 50%. Moreover, combining GA with FPG or 2hPG significantly improved the identification efficacy for TITR > 50%. These findings reinforce the relationship between traditional glycemic indicators and CGM metrics, providing valuable insights for glycemic assessment in the absence of CGM.

As CGM has gained progressively wider clinical application, new metrics derived from this technology have emerged, including TIR (3.9–10.0 mmol/L). A growing body of evidence has demonstrated close associations between TIR and diabetes‐related outcomes [25, 26]. Recently, a more stringent glycemic metric TITR (3.9–7.8 mmol/L) has garnered increasing attention and has been suggested as an endpoint for CGM‐related clinical trials [6]. TITR reflects a stricter glycemic control target, more closely aligning with the physiological blood glucose levels of healthy individuals [27]. It is also considered a useful indicator for predicting the future risk of diabetes‐related complications [8, 28, 29]. A retrospective analysis of real‐world data from 22 006 CGM users collected between 2014 and 2021 revealed that for patients targeting lower average glucose and HbA1c targets, such as those aiming for type 2 diabetes remission or near‐normal glycemia in type 1 diabetes—TITR was more sensitive than TIR to changes in average glucose and glycemic variability [7]. As mentioned above, the study by Petersson et al. [22] found that TITR of 50% was equivalent to HbA1c of 6.5%. Additionally, a study involving patients using advanced hybrid closed‐loop technology reported that over 70% of participants achieved a TITR > 50%, and more than 90% met the target of TITR > 45% [30]. Another cross‐sectional study of 56 children and adolescents showed that 87% of the participants achieved a TITR exceeding 50% [31]. These findings support the recommendation to adopt TITR > 50% as a standard for glycemic control targets [32].

Previous studies on the relationship between traditional glycemic indicators and TITR or TIR are relatively limited [11, 22, 33, 34]. A multicenter study involving 133 children and adolescents with type 1 diabetes found a nonlinear association between HbA1c and TITR over the previous 60 days (*R* ^2^ = 0.69) [22]. Another study conducted in 854 children and adolescents with type 1 diabetes used multiple linear regression analysis, showing a negative correlation between HbA1c and TITR (*R* ^2^ = 0.56) [34]. Recently, an analysis from a 24‐week prospective study found that among 34 patients with type 1 or type 2 diabetes, GA was the most accurate glycemic marker for predicting TIR within 8 weeks compared to fructosamine and HbA1c [11]. The GA cutoff for identifying TIR > 70% was 17.5%, highlighting its utility in assessing glycemic changes during periods without CGM [11]. In comparison to previous studies, our study included 310 well‐controlled patients with type 2 diabetes whose treatment regimens remained unchanged. The results showed a GA cutoff of 17.4% corresponding to a TIR of 70%, consistent with earlier findings. Furthermore, this study is the first to explore the relationship between GA and TITR in patients with type 2 diabetes, revealing a significant negative correlation.

While HbA1c and GA demonstrated comparable AUC values for detecting TITR > 50% and TIR > 70% (Figure 1), their distinct biological characteristics and monitoring timeframes translate to complementary roles in clinical practice. HbA1c, as the gold standard for long‐term glycemic control (8–12 weeks), remains pivotal for routine evaluations in stable patients, aligning with guideline‐recommended 6‐month reassessments. In contrast, GA is sensitive to short‐term fluctuations (2–3 weeks) which makes it particularly valuable for scenarios requiring rapid feedback, such as post‐therapeutic adjustments or acute metabolic stress. Notably, our findings highlighted that GA had a unique advantage when combined with FPG or 2hPG, significantly improving specificity and AUC for GA, whereas HbA1c showed no such benefit. This supports GA's utility in contexts requiring simultaneous assessment of acute glycemic variability and intermediate‐term control, especially in populations with comorbidities like anemia, where HbA1c may be confounded. These insights align with the ADA guideline's emphasis on individualized monitoring strategies, positioning GA as a complementary tool to HbA1c for tailored glycemic management. Moreover, from a clinical application perspective, GA can be measured alongside glucose and other biochemical markers in a single blood sample, allowing for a streamlined approach to assessing TITR during non‐CGM periods. A previous study by our team demonstrated that combining GA and FPG significantly enhanced the sensitivity of diabetes screening, eliminating the need for OGTT in 76% of patients [35]. Similarly, a study by Selvin et al. [36], involving a 25‐year follow‐up of 12 199 participants, showed that measuring FPG and HbA1c simultaneously from a single blood sample provided strong predictive power for diabetes development. These findings further underscore the clinical value of combined testing.

Notably, this study found that the GA cutoffs for identifying TIR > 70% and TITR > 50% were consistent, highlighting the relationship between TIR and TITR. Previous studies have shown a nonlinear relationship between TIR and TITR, where the TITR/TIR ratio increases as TIR rises. This relationship also varies with glycemic variability and TBR [37]. Our preliminary analysis demonstrated a nonlinear correlation between TIR and TITR, with a TITR of approximately 40% corresponding to a TIR of 70% (Figure S3). This finding aligns with prior studies, which observed that TITR was on average 20%–25% lower than TIR across different diabetes cohorts and insulin therapy modalities [38]. However, the GA cutoff corresponding to TITR > 50% aligns with that of TIR > 70%, which contradicts the nonlinear thresholds of TITR and TIR. This apparent discrepancy may be attributed to distinct statistical frameworks: the GA threshold was derived from ROC curve analysis maximizing the Youden index (sensitivity/specificity balance) to broadly capture suboptimal glucose control, whereas the nonlinear association was modeled using generalized additive methods to characterize their physiological interdependence. While separate GA cutoffs could theoretically optimize prediction for isolated TIR or TITR thresholds, the unified value enhances clinical utility by identifying individuals requiring intervention, regardless of their specific dysglycemia phenotype.

The strengths of this study included a relatively large sample of type 2 diabetes patients with unchanged treatment regimens and the first exploration of the relationship between GA and TITR. Additionally, the combination of GA with fasting and postprandial glucose further improved the efficacy of identifying TITR targets. However, several limitations should be noted. Firstly, this study was a single‐center observational study, and its findings need to be validated in larger, multicenter studies. Secondly, previous research has demonstrated that when CGM is worn for 5 days or longer, TIR strongly correlates with the full 3‐month CGM results (*R* ^2^ > 0.80) [15]. In this study, the median CGM wear time was 6.0 days, although shorter than the 14‐day guideline recommendation, which is still considered reliable. Thirdly, the participants in this study were inpatients with type 2 diabetes who followed a standard diet during the CGM monitoring period. This hospitalized context may involve a more complex patient population, which could introduce potential selection bias. Therefore, the generalizability of our findings to other populations with diabetes, or those in outpatient or community settings, may be limited. Furthermore, factors affecting GA, such as those analyzed in the sensitivity analysis, were also taken into consideration. Previous studies [3] have shown that the influence of increased body fat on GA levels may be mediated by mechanisms related to fat mass and visceral adipose tissue. Clinically, it is important to recognize that in individuals with higher body fat or central obesity, GA may underestimate actual blood glucose levels.

This study confirmed a negative correlation between GA and both TITR and TIR in well‐controlled type 2 diabetes with stable treatment regimens. The optimal GA cutoff for identifying TITR > 50% or TIR > 70% was found to be 17.4%, demonstrating the potential utility of traditional glycemic indicators for glucose assessment during non‐CGM periods. Future large‐scale studies are needed to further investigate the relationship between GA and different TITR target thresholds.

## Author Contributions

X.M., J.L., and J.Z. conceived and designed the study. J.N. and W.H. contributed to data analysis and manuscript preparation. Y.W., J.Y., W.L., and Y.W. contributed to data collection. X.M., J.L., and J.Z. contributed to the interpretation of data and the revision of the manuscript. All authors read and approved the final manuscript.

## Ethics Statement

The study and the analysis plan were approved by the Research Ethics Committees of Shanghai Sixth People's Hospital Affiliated to Shanghai Jiao Tong University School of Medicine.

## Consent

We have obtained informed consent from all participants.

## Conflicts of Interest

The authors declare no conflicts of interest.

## Supporting information


**Data S1.** Supporting Information.

## Data Availability

Restrictions apply to the availability of some or all data generated or analyzed during this study to preserve patient confidentiality or because they were used under license. The corresponding author will, on request, detail the restrictions and any conditions under which access to some data may be provided.
