# Supplementary material for: The Relationship Between Glycated Albumin and Time in Tight Range in Type 2 Diabetes
Source: J Diabetes. 2025 Mar 26;17(3):e70073. doi: 10.1111/1753-0407.70073 (PMC11938112; doi:10.1111/1753-0407.70073)
Supplement: Supplementary file 1 — Data S1. Supporting Information. [file JDB-17-e70073-s001.docx]

**Supplemental Table 1. Effectiveness of GA, HbA1c and their combinations with FPG or 2hPG in identifying TITR > 50% or TIR > 70% (N=251)**

|  | **AUC** | **Cutoff** | **Sensitivity (%)** | **Specificity (%)** | **PPV (%)** | **NPV (%)** | **NRI (95%CI)** | **IDI (95%CI)** |
| --- | --- | --- | --- | --- | --- | --- | --- | --- |
| **TITR > 50%** |  |  |  |  |  |  |  |  |
| GA | 0.685 (0.600, 0.769) | 17.4% | 82.76 | 47.92 | 87.05 | 39.66 | reference | reference |
| HbA1c | 0.734 (0.655, 0.812) | 6.8% | 72.41^b^ | 66.67^c^ | 90.18 | 36.36 | 0.49 (0.19-0.78) ^d^ | 0.03 (−0.02-0.08) |
| FPG | 0.755 (0.674, 0.835) | 6.6 mmol/L | 83.74 | 64.58 | 90.91 | 48.44 | 0.42 (0.12-0.72) ^d^ | 0.09 (0.01-0.17) ^e^ |
| 2hPG | 0.749 (0.675, 0.823) | 8.6 mmol/L | 58.62^b^ | 81.25^c^ | 92.97 | 31.71 | 0.18 (−0.12-0.48) | 0.03 (−0.03-0.10) |
| GA + FPG | 0.786 (0.716, 0.855) ^a^ |  | 81.28 | 62.50 | 90.16 | 44.12 | 0.77 (0.48-1.05) ^d^ | 0.13 (0.07-0.19) ^e^ |
| GA + 2hPG | 0.771 (0.701, 0.841) ^a^ |  | 74.88 | 72.92^c^ | 92.12 | 40.70 | 0.44 (0.14-0.74) ^d^ | 0.07 (0.03-0.11) ^e^ |
| HbA1c + FPG | 0.782 (0.716, 0.848) |  | 73.89 | 70.83^c^ | 91.46 | 39.08 | 0.63 (0.34-0.92) ^d^ | 0.12 (0.04-0.19) ^e^ |
| HbA1c + 2hPG | 0.783 (0.716, 0.849) ^a^ |  | 74.38 | 72.92^c^ | 92.07 | 40.23 | 0.42 (0.12-0.72) ^d^ | 0.08 (0.02-0.15) ^e^ |
| **TIR > 70%** |  |  |  |  |  |  |  |  |
| GA | 0.754 (0.643, 0.864) | 17.4% | 80.79 | 63.64 | 95.85 | 24.14 | reference | reference |
| HbA1c | 0.764 (0.657, 0.870) | 6.9% | 73.36^b^ | 77.27 | 97.11 | 21.79 | 0.09 (−0.35-0.53) | −0.01 (−0.07-0.04) |
| FPG | 0.595 (0.452, 0.739) | 6.6 mmol/L | 76.86 | 50.00 | 94.12 | 17.19 | −0.41 (−0.84-0.02) | −0.05 (−0.12-0.03) |
| 2hPG | 0.675 (0.560, 0.790) | 8.9 mmol/L | 55.90^b^ | 77.27 | 96.24 | 14.41 | −0.35 (−0.78-0.08) | −0.05 (−0.11-0.01) |
| GA + FPG | 0.757 (0.644, 0.870) |  | 80.35 | 63.64 | 95.83 | 23.73 | 0.28 (−0.16-0.71) | 0.02 (−0.02-0.06) |
| GA + 2hPG | 0.769 (0.671, 0.868) |  | 61.57^b^ | 86.36 | 97.92 | 17.76 | 0.14 (−0.29-0.58) | 0.01 (−0.02-0.03) |
| HbA1c + FPG | 0.762 (0.655, 0.868) |  | 65.50^b^ | 81.82 | 97.40 | 18.56 | 0.23 (−0.21-0.66) | −0.00 (−0.07-0.06) |
| HbA1c + 2hPG | 0.775 (0.678, 0.871) |  | 75.55 | 77.27 | 97.19 | 23.29 | 0.05 (−0.38-0.49) | −0.06 (−0.06-0.05) |

Abbreviations: 2hPG, 2-hour plasma glucose; AUC, area under curve; CI, confidence interval; FPG, fasting plasma glucose; GA, glycated albumin; HbA1c, glycated hemoglobin A1c; IDI, integrated discrimination improvement; NRI, net reclassification improvement; TIR, time in range (3.9-10.0 mmol/L); TITR, time in tight range (3.9-7.8 mmol/L). ^*^*P* values for comparisons of different models in identifying TITR > 50% or TIR > 70% relative to GA.

^a^*P*<0.05 for comparisons of AUC between different models in identifying TITR > 50% or TIR > 70% compared to GA as the reference indicator.
^b^*P*<0.05 for comparisons of sensitivity between different models in identifying TITR > 50% or TIR > 70% compared to GA as the reference indicator.
^c^*P*<0.05 for comparisons of specificity between different models in identifying TITR > 50% or TIR > 70% compared to GA as the reference indicator.

^d^*P*<0.05 for comparisons of NRI between different models in identifying TITR > 50% or TIR > 70% compared to GA as the reference indicator.

^e^*P*<0.05 for comparisons of IDI between different models in identifying TITR > 50% or TIR > 70% compared to GA as the reference indicator.

**Supplemental Figure 1. Flow chart of the study**


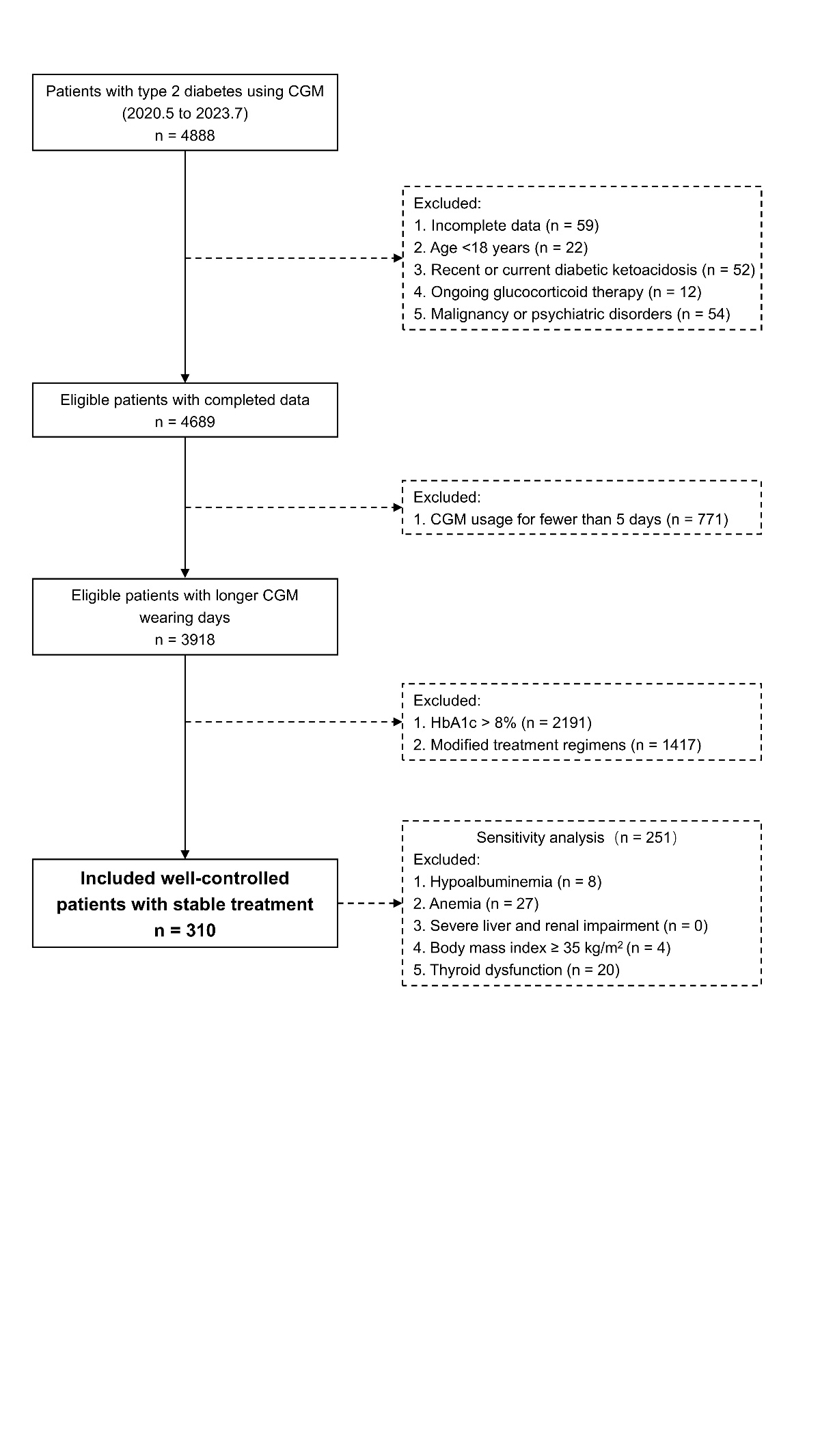


**Supplemental Figure 2. ROC curves of GA, HbA1c and their combinations with FPG or 2hPG in identifying TITR > 50% or TIR > 70% (N=251)**

**
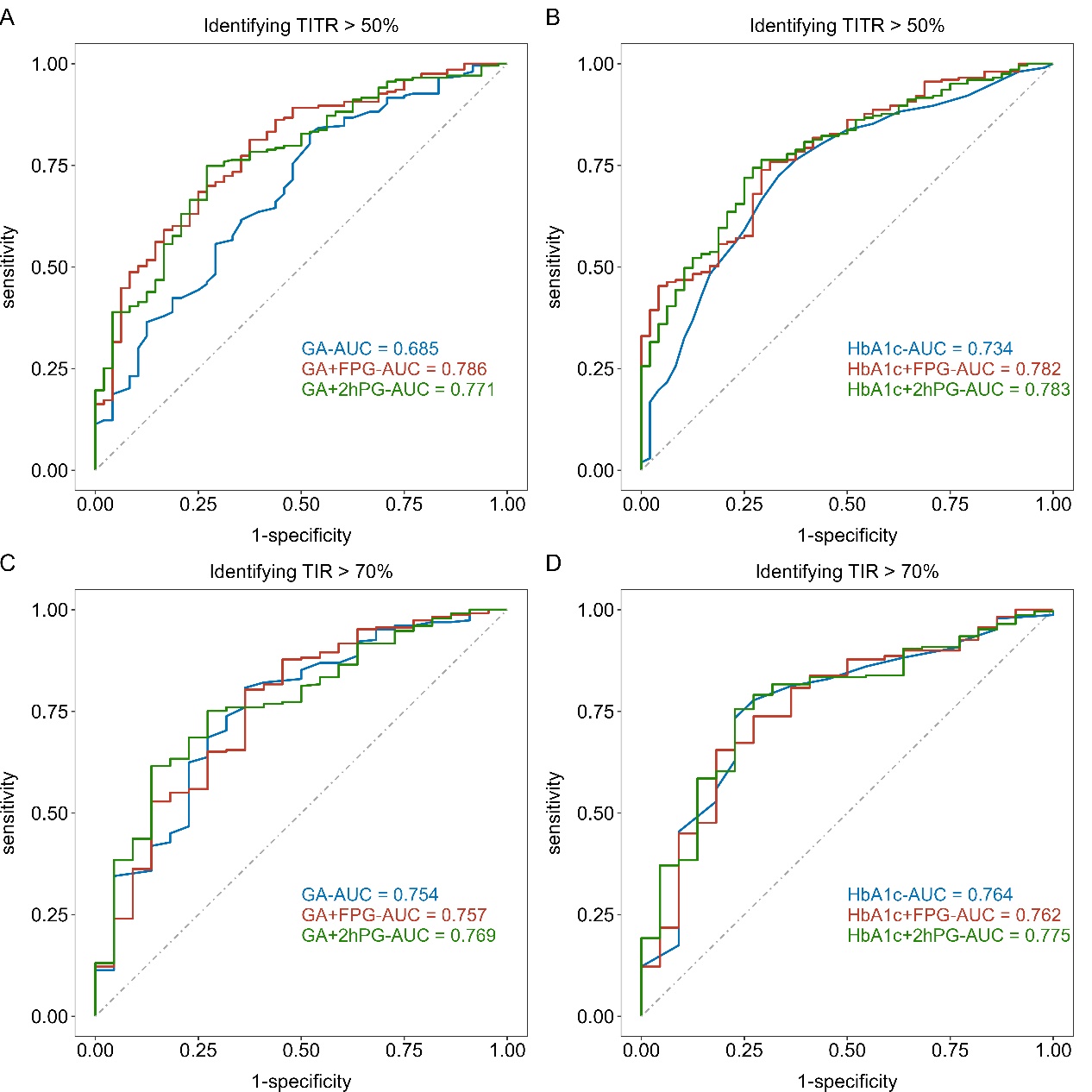
**

Abbreviations: 2hPG, 2-hour plasma glucose; FPG, fasting plasma glucose; GA, glycated albumin; HbA1c, glycated hemoglobin A1c; ROC, receiver operating characteristic; TIR, time in range (3.9-10.0 mmol/L); TITR, time in tight range (3.9-7.8 mmol/L).

**Supplemental Figure 3. Relationship between TITR and TIR**

**
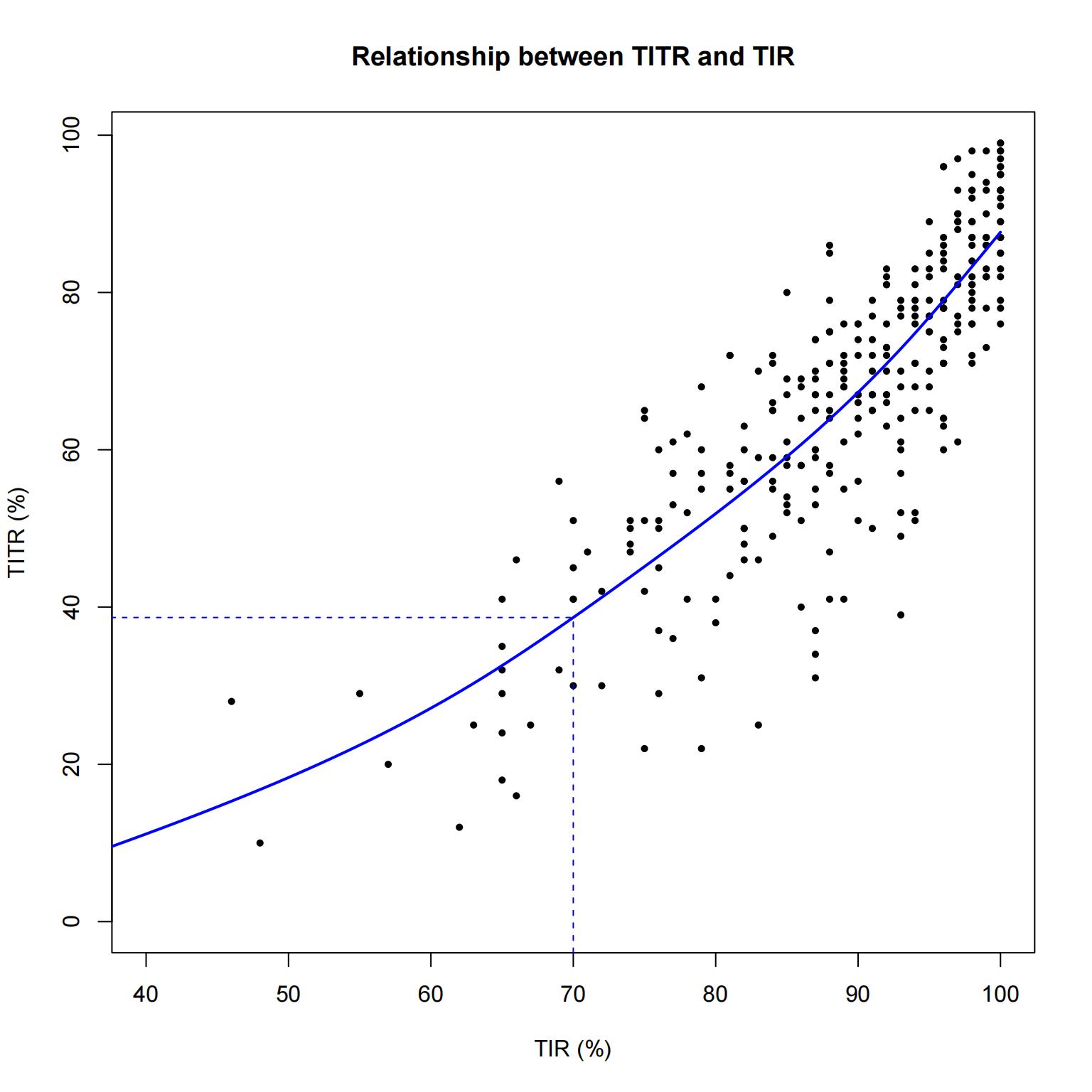
**

Abbreviations: TIR, time in range (3.9-10.0 mmol/L); TITR, time in tight range (3.9-7.8 mmol/L).
